# Individualized Interactive Instruction: A Guide to Best Practices from the Council of Emergency Medicine Residency Directors

**DOI:** 10.5811/westjem.2018.12.40059

**Published:** 2019-02-11

**Authors:** Molly Estes, Puja Gopal, Jeffrey N. Siegelman, John Bailitz, Michael Gottlieb

**Affiliations:** * Loma Linda University, Department of Emergency Medicine, Loma Linda, California; † University of Chicago, Department of Emergency Medicine, Chicago, Illinois; ‡ Emory University, Department of Emergency Medicine, Atlanta, Georgia; § Northwestern University Emergency Medicine, Feinberg School of Medicine, Chicago, Illinois; ¶ Rush University Medical Center, Department of Emergency Medicine, Chicago, Illinois

## Abstract

Over the last several years, there has been increasing interest in transitioning a portion of residency education from traditional, lecture-based format to more learner-centered asynchronous opportunities. These asynchronous learning activities were renamed in 2012 by the Accreditation Council for Graduate Medical Education (ACGME) as individualized interactive instruction (III). The effectiveness and applicability of III in residency education has been proven by multiple studies, and its routine use has been made officially acceptable as per the ACGME. This article provides a review of the current literature on the implementation and utilization of III in emergency medicine residency education. It provides examples of currently implemented and studied III curricula, identifies those III learning modalities that can be considered best practice, and provides suggestions for program directors to consider when choosing how to incorporate III into their residency teaching.

## BACKGROUND

One of the most recent trends in medical education is the transition from traditional didactics (i.e., lecture-based classroom teaching) to online learning modules, collectively referred to as asynchronous learning. Over the last several years, asynchronous learning has been shown to be a successful learning style for many learners. For example, Liu and colleagues performed a meta-analysis of what the authors termed “blended learning” (i.e., the combination of traditional teaching methods with asynchronous learning) throughout all health professional learners. Their review found that blended learning consistently performed better than no intervention and that it did not perform inferiorly to traditional “non-blended” learning.^1^ A host of additional data exists, demonstrating that learners prefer smaller learning environments^2^ and that these methods can address the challenge of teaching physician self-assessment and fostering the practice of lifelong learning.^3^

It is known that there is a broad range of the use of asynchronous learning across the field of medicine. Looking specifically at resident training, a survey of internal medicine program directors (PD) revealed that out of the 214 responding programs, 71.5% used asynchronous learning sometimes, somewhat often, or very often.^4^ Examples of asynchronous learning curricula can be found in nearly every medical area and specialty, from a pediatrics gastroenterology subspecialty rotation,^5^ microsurgery competencies in plastic surgery,^6^ and radiology residents receiving more real-time feedback on radiographic reads,^7^ to journal club for general surgery.^8^ There are examples for the training of fellows^9^ and faculty.^10^ There are even examples of all learners, laypeople and medical professionals, participating in a basic life support class^11^ and for interprofessional learners from all levels of training and fields participating in teamwork training.^12^

The early 2000s to 2010s saw a unique challenge to residency programs specifically as Free Open Access Meducation (FOAM) resources increased exponentially.^13^ Anecdotal evidence at that time suggested that residents were using these resources for their own asynchronous education, with or without residency program oversight. Programs faced the decision to either begin vetting and incorporating these resources into their curricula or to maintain a more traditional didactic approach. Questions were raised whether time spent in asynchronous learning could even be counted as part of Accreditation Council for Graduate Medical Education (ACGME) required didactic time.

In 2008, the Council of Emergency Medicine Residency Directors (CORD) in conjunction with a task force from the Residency Review Committee for Emergency Medicine (RRC-EM) set out to critically evaluate the ACGME EM Program Requirements specifically pertaining to educational conferences. One of the suggestions from that task force was for residency programs to actively consider incorporating asynchronous learning as an educational tool.^3^ Not long after the task force’s recommendations, the RRC-EM published criteria allowing up to 20% of conference didactic time to be spent in asynchronous learning, which was renamed individualized interactive instruction (III).^14^ A subsequent publication from the same group further defined specific requirements of a valid III program (see section on “Cautions of Implementation”).^15^

Since then, there has been increasing research into how and which aspects of EM residency teaching can be transitioned to III.^16^ Some programs have applauded it as the way of the future,^17^–^18^ while others have advised caution in implementation.^19^–^20^ Multiple ideas have been published on how to incorporate III such as flipped classroom,^21^ journal article discussion boards,^22^ or a series of varied online learning tasks.^23^ Comprehensive databases have emerged offering vetted sources, centralized information, and access to experts.^24^

Surveys have shown extensive utilization of III among residents,^25^ as well as significant incorporation into EM training programs.^26^–^27^ A survey by Waxman and colleagues in 2014 showed that 63% of programs were incorporating III into residency training; however, they noted there were significant variations in the structure of the curricula. Of the 37% that were not using III, 71% had concerns related to the understanding and implementation of III within the ACGME/RRC-EM criteria.^26^ The purpose of this article is to provide a review of the current literature on III and best practices recommendations for programs to consider as they refine their already-existing III curricula or implement a curriculum for the first time.

## APPRAISAL OF THE LITERATURE

This article is the second in a series of best practice reviews from the CORD Best Practices Subcommittee. The first three authors performed a search of PubMed for articles published from inception to March 31, 2018, using the same keywords “asynchronous learning” and “individualized interactive instruction.” Bibliographies of all relevant articles were reviewed for additional studies. The search authors screened articles to evaluate for any that addressed the specific topics of implementation and utilization of III curricula within the field of EM.

The search yielded a total of 664 articles, of which 19 were deemed to be directly relevant to EM and for inclusion in this review. When supporting data were not available, recommendations were made based upon the authors’ combined experience and consensus opinion. Prior to submission, the manuscript was reviewed by the entire CORD Best Practices Subcommittee. It was additionally posted to the CORD website for two weeks for general feedback and review from the entire CORD community.

## CURRENT USES OF III IN EMERGENCY MEDICINE

In 2015, the CORD III Task Force performed an updated survey of PDs of ACGME-accredited EM residency programs on their current use of III (unpublished data). Of the 77 unique programs that responded (approximately 46% response rate), 74% reported incorporating III into their programs. More four-year format programs used III than three-year programs (91% compared to 67%). Programs implementing III were divided among those who offered either four or five hours of synchronized didactics weekly, or some variation thereof. Of those who reported not using III, the most cited rationale was an unclear definition of what constituted III. Other programs were concerned about compliance or the resources required for implementation. Offerings for III credit were quite diverse. Many programs offered online learning modules, FOAM resources, and board review sessions for III credit. Some used simulation, journal club, and attendance at national or regional meetings. This survey shows that although there is a high rate of utilization of III among programs, there still remains a wide variation in qualifying activities.

While there is a significant amount of literature on the importance and acceptance of III as a learning tool, no standard or consensus method of implementation currently exists in EM. In addition, there is a dearth of information (only the single survey as described above) in the published literature as to how individual EM residency programs specifically implement III. And there is significant variation among programs based on qualitative preliminary surveys. Some research even suggests that III may not be an adequate replacement for all of the didactics in a traditional curriculum, specifically for novice learners, concerns namely being their ability to identify specific knowledge gaps and their need to have adequate expert oversight to ensure true knowledge acquisition and retention.^20^ Several publications in recent years highlight examples of how EM residency programs nationwide have and are using III; some selected examples are discussed below.

Wray and colleagues implemented an III curriculum in 2013 and measured the effect on in-training exam (ITE) scores. Faculty and chief residents created four modules per month, each designed to be completed in less than one hour. Educational content included journal articles, audio and video lectures, podcasts, links to FOAM resources, and modules linked to quizzes. Residents were required to complete these modules, and their progress was monitored in addition to ITE scores. The group found that despite the decrease in traditional conference hours, time now allotted to III, there was no negative impact on resident ITE scores.^28^

Pensa and colleagues created a digital course for residents in 2014 and surveyed residents to assess satisfaction. The program educational material was curated by faculty from various FOAM/digital resources, and participation was optional. The modules included an assignment page with the content; a discussion page, which was a mandatory component of the module and allowed for learners to post queries and for faculty members to answer questions; and a multiple-choice quiz page for assessment. Thirty-three of 48 residents participated in the survey in the first year and appeared overall to find the course useful, although there were significant variations in time spent participating in the course both among residents as well as faculty. The biggest barrier to participation identified by residents was lack of time.^29^

Kornegay and colleagues developed an III curriculum implemented during the 2011–2012 academic year. Faculty members identified gaps in the pre-existing synchronous curriculum and topics better suited for independent learning and then developed a web-based platform consisting of curated content and an evaluation component, namely a reflective writing assignment or quiz. Of responding residents, about 80% were satisfied, very satisfied, or extremely satisfied with the new modality. The group also analyzed conference attendance and ITE scores and found that postgraduate year (PGY)-1 resident attendance rate significantly improved from the prior year (85% vs 62% mean), although other curricular changes in the program (e.g., small group-based learning, interactive case-based conferences, and changes in off-service rotations) may have also enhanced participation. There was no statistically significant difference in mean ITE scores pre- and post-intervention. Faculty reported a time commitment of about four to eight hours per month, which was comparable to the time spent to prepare one hour of instruction for weekly conference pre-intervention.^30^

Kothari and colleagues designed an III curriculum based on Academic Life in Emergency Medicine (ALiEM)’s popular Approved Instructional Resources (AIR) series. The AIR series curates FOAM content from the top 50 open-access EM and critical-care blog and podcast sites, provides associated core teaching points and multiple-choice questions for residents, and tracks resident participation to provide residency PDs with resident progress.^24^ Kothari and colleagues then implemented a second component to their III curriculum, which consisted of two high-impact journal articles selected by faculty on a monthly basis. The group found that introduction of the III did not negatively affect residency educational conference; attendance across all PGY levels was comparable to the year before.^31^

Other innovative strategies and formats to implement III in EM have been centered upon discrete, focused topic areas within the larger EM curriculum, such as pediatrics,^32^–^33^ palliative and end-of-life care,^34^ and disaster medicine.^35^ Commonalities exist among these examples, namely facilitators’ deliberate choosing of either a specific asynchronous learning program or a specific topic to be taught using asynchronous learning depending on their program’s needs.

## BEST PRACTICE RECOMMENDATIONS

1. III should be used cautiously with the novice learner.
2. When deciding to develop or implement an III curriculum, first identify gaps in the current curriculum or those topics that may be best transitioned to an III format. This is likely to vary between programs.
3. A combination of available III (e.g., online blogs, podcasts, and journal articles) seems to attract a greater number of residents to participate, likely as this variety addresses a broader span of individual learning preferences.
4. Transition to III does not seem to negatively affect resident ITE scores or weekly conference attendance rates.

## CAUTIONS OF IMPLEMENTATION

The ACGME policy statement on the use of III within EM residency education is very strict as to the criteria that must be met for an activity to be considered III. Given that up to 20% (one out of every five hours) of previously considered core curriculum time can now be spent as III, there may be a natural inclination among programs to begin to cut back on planned, traditional educational activities. This is a fallacy, and there are several ways that implementation of III can go wrong (Table).^15^ Below are listed some common pitfalls encountered when implementing III.

### Independent Reading and Use of Question Banks

The ACGME places particular emphasis on any potential III being a planned activity that is tailored for the individual’s level of learning. Resident-directed reading is not considered a planned activity. Additionally, independent use of a question bank is not directed to the individual’s particular needs, even if the astute resident is choosing specific topics to review. Faculty may choose a specific reading or set of questions to include as a part of III, but these by themselves do not qualify.

### Resident Attestations of Completion

An attestation of completion of an III activity is not considered to be adequate enough to prove resident participation. There must be a separate, tangible source of evaluation. Tracking quiz completion/participation after an online module or required reading would provide ample proof of activity completion, just as a sign-in sheet before a simulation does the same.

### Audio, Video, or Podcasts

These learning methods are considered to be passive learning, and use of them alone does not qualify as III. However, they can be combined with other learning modalities, such as a particular question set from an online question bank, to include an active component.

### Monitoring for Effectiveness

At the time of implementation of the chosen curricula, PDs must have a plan for how they will go about tracking the effectiveness of the III program. This can take many different forms: use of periodic review quizzes; objective clinical performance; test scores on the ITE, etc. However, this type of evaluation must be planned over several generations of residents to account for individual class variation and ensure the III program itself is not causing knowledge gaps. Regular check-ins with residents to ensure their continued perspective of the curricula as beneficial are also recommended.

## BEST PRACTICE RECOMMENDATIONS

1. Before designing or implementing an III curriculum, carefully review the ACGME criteria to ensure compliance.
2. Resident-driven use of question banks does not meet III criteria.
3. An attestation of completion does not meet III criteria for participation.
4. Use of passive learning methods alone (e.g., podcasts) does not meet III criteria.
5. Regular curriculum assessment is essential to ensure adequate instructional merit and continued benefit to resident learning.

## OPTIONS FOR III ACTIVITIES

Several best practices have emerged from surveying EM PDs who have implemented III, both with respect to high quality, effective educational programming and compliance with RRC-EM regulations.^27^

### Simulation

Simulation activities easily satisfy the requirements of III. They can provide an individual resident the opportunity for self-directed work on a particular area of improvement with direct faculty supervision and immediate feedback. These work best when a resident identifies a particular case, topic, or procedure on which he or she would like to focus.

### Online Resources

A wealth of freely accessible material is available for III learning via podcasts, blogs, and online modules. PDs need to creatively consider how they will allow for the use of such material for III while maintain compliance with RRC regulations. Additionally, faculty must take care to appropriately vet all resources to ensure credibility and academic rigor.^36–37^ Perhaps the most widely adopted single resource is the ALIEM-AIR Series,^24^ which (as of its 2016 publication) has been implemented in 65 programs. This group rigorously selects the highest quality online resources, as judged by EM faculty, provides a quiz for an evaluative component, and allows for online discussion. Individual PDs are able to monitor both the modules as well as their residents’ participation. Other best practices include discussion sessions with a faculty lead about a particular podcast or blog post.

### National/Regional Conferences

Attendance at specialty society meetings offers many learning opportunities. To rise to the level of III and meet the criteria set forth by the ACGME, programs have instituted a number of policies for such activities. Monitoring participation and faculty oversight are key areas of concern, and can be addressed by checking in with faculty who are also attending or presenting at a particular session. Some programs require discussion or written assignments following the session or conference.

### Question Banks

Many question banks are available online and in print for residents’ use in preparing for standardized tests. While answering questions alone does not meet criteria for III (see “Cautions of Implementation” above), reviewing specific questions missed or themes with a faculty member would be acceptable.

### Other Opportunities

Multiple other activities are in use in EM programs for III including journal clubs, research and teaching activities, oral boards practice, and many others.

## BEST PRACTICE RECOMMENDATIONS

1. When designing an III curriculum, many options for learning activities are available to be included: simulation, online resources, national/regional conferences, question banks with faculty oversight, etc.
2. When choosing online sources, take care to ensure credibility and academic rigor, scoring methods exist and can be used to assess these factors.

## LIMITATIONS

While all attempts have been made to create an inclusive review of the current use of III in EM residency education, limitations must be acknowledged. In the identification of pertinent articles for inclusion, although multiple search terms were used and bibliographies cross-referenced, it is possible that some articles may not have been identified by the current review. We chose articles based on their primary relevance to the field of EM; thus, our analysis was not intended to be an expansive review of the history of the use of III or its current use in other medical fields or specialties. In the absence of data, every effort was made to make conservative recommendations based on the authors’ experience and expertise as educators in the field of EM and, although a potentially limiting factor, these opinions were available for review by the entire CORD Best Practices Subcommittee prior to publication.

The primary limitation to this data analysis was the relative paucity of data available on the direct implementation or utilization of full III curricula within EM residency programs. Multiple sources have supplied information pertaining to the use of specific, topic-based curricula, but few show analysis of a more extensive use of III as might pertain to what can be considered a core curriculum.

## CONCLUSION

This article provides a review of the literature currently available on the implementation and use of III in emergency medicine residency education. It can be said conclusively that III has been proven to be an accepted part of modern residency education. Preliminary data suggest that III may very well augment resident learning without negatively affecting standardized testing scores or resident participation in other traditional didactics. Care must be given to choose the appropriate learning level of the resident and ensure ACGME compliance with curricular activities. However, despite multiple sources of curricula options, there remains a paucity of information regarding the effectiveness of specific III as it pertains to resident knowledge acquisition and retention. More research is needed to further refine what we determine to be gold standard III modalities. Until then, it is the authors’ intention that readers will be more aware of the ACGME guidelines and the III options that exist in order to avoid the potential pitfalls of implementation at their home institutions.

**CORD Best Practice Committee 2017–18**

**Michael Gottlieb, MD – Co-Chair**
  Rush University Medical Center
**John Bailitz, MD – Co-Chair**
  Northwestern University, Feinberg School of Medicine
**Jeremy Branzetti, MD**
  New York University/Bellevue
**Richard Byyny, MD**
  Denver Health Medical Center
**Molly Estes, MD**
  Loma Linda University
**Puja Gopal, MD**
  University of Illinois in Chicago
**Albert Kim, MD**
  Washington University in Saint Louis
**Andrew King, MD**
  The Ohio State University
**Melissa Parsons, MD**
  University of Florida - Jacksonville

## Figures and Tables

**Table:** ACGME criteria for III.^15^

The program director must monitor resident participation. There must be an evaluation component. There must be faculty oversight. The activity must be monitored for effectiveness.

*ACGME,* Accreditation Council for Graduate Medical Education; *III*, individualized interactive instruction.
